# Genetic Indicators of Performance Resilience in Barrel Racing Quarter Horses

**DOI:** 10.1002/age.70158

**Published:** 2026-07-06

**Authors:** Mário Luiz Santana, Annaiza Braga Bignardi

**Affiliations:** ^1^ Grupo de Melhoramento Animal de Mato Grosso (GMAT), Instituto de Ciências Agrárias e Tecnológicas (ICAT) Universidade Federal de Rondonópolis (UFR) Rondonópolis Brazil

**Keywords:** career earnings, genetic parameters, genotype by environment interaction, speed, sport horse, stability

## Abstract

Resilience, defined as the capacity to maintain consistent performance under variable conditions, has emerged as a relevant breeding objective in livestock systems, yet remains largely unexplored in equine populations. The objectives of this study were to (1) derive indicators of resilience from longitudinal barrel racing time (BRT) records in Brazilian Quarter Horses, (2) estimate genetic parameters for these indicators, and (3) quantify their genetic relationships with BRT and accumulated career earnings (ACE). The dataset comprised BRT records from barrel racing events held between 2010 and 2024, involving 10 665 horses. Resilience indicators were derived from short‐term deviations around an expected performance trajectory after removing systematic effects and persistent horse‐ and rider‐specific performance levels. Three indicators were evaluated: median absolute deviation (MAD), logarithm of the variance of deviations (LNVAR), and proportion of positive deviations (PPOS). Posterior mean heritability estimates for the resilience indicators were low (0.06 to 0.09), but still indicated exploitable additive genetic variation. Genetic correlations revealed a near‐complete overlap between MAD and LNVAR (0.987), while PPOS captured partially distinct genetic information. All resilience indicators showed favorable genetic associations with ACE (mean −0.59) and positive associations with BRT (mean 0.44), indicating that genetically more resilient horses tended to perform faster and achieve higher economic returns. Therefore, resilience indicators derived from longitudinal performance records captured genetically meaningful variation related to performance consistency and economic success. These indicators complemented traditional performance traits and provided a scalable framework for incorporating resilience and long‐term regularity into genetic evaluations of sport horses.

## 1 Introduction

In contemporary animal breeding programs, resilience represents an animal's capacity to sustain productive function under environmental challenges or demonstrate rapid recovery after stress events (Berghof, Poppe, et al. 2019; Colditz and Hine 2016). From a quantitative genetics perspective, resilience is not a directly observable trait but a latent biological property that can be inferred from patterns of phenotypic variation expressed over time (Friggens et al. 2024; Taghipoor et al. 2023). The increasing availability of longitudinal and high‐frequency records has substantially expanded opportunities to define resilience indicators based on within‐individual deviations around an expected level of performance, enabling their integration into genetic evaluation frameworks.

Genetic resilience is closely connected to key functional traits that determine the sustainability and profitability of animal production systems. Across livestock species, research has established associations between reduced performance variability and favorable outcomes including better health indicators, superior reproductive success, and extended productive lifespan (Chen et al. 2023; Poppe et al. 2020; Rodrigues et al. 2025). These relationships highlight that resilience captures information that is partly independent from average performance levels and complements traditional selection objectives. Consequently, incorporating resilience‐related traits into breeding goals can be viewed as a strategy to improve animal adaptability, reduce management costs, and enhance overall system efficiency.

The relevance of resilience has become even more pronounced as breeding programs increasingly operate under heterogeneous and challenging environments. Climate variability, fluctuating management conditions, and intensified production systems expose animals to a wide range of micro‐ and macro‐environmental stressors (Berghof, Poppe, et al. 2019; Poppe et al. 2021). Under such conditions, selection exclusively for high mean performance may lead to increased sensitivity to disturbances (Carabaño et al. 2021; Freitas et al. 2021; Santana et al. 2015), whereas selection for resilience‐related attributes can contribute to more stable and predictable outcomes across time and environments.

Although resilience concepts are highly relevant to equine athletics, the genetic evaluation of performance stability in sport horses remains limited. Related approaches have been explored in horses through heteroscedastic or canalization models, which allow the mean of a trait and its environmental variability to be evaluated jointly (Cervantes et al. 2020; Poyato‐Bonilla et al. 2021). These studies demonstrated that variability‐related traits can be genetically modeled in equine populations, particularly for competition rank and morphology. However, they did not address resilience indicators derived from longitudinal deviations in athletic performance.

This gap is relevant because competitive success depends not only on peak performance but also on the ability to express consistent results across repeated events, seasons, and competitive contexts. Moreover, the feasibility of incorporating resilience‐related indicators into genetic improvement programs, as well as their genetic relationships with competitive performance and economic success, remains unknown in sport horses. Barrel racing in Quarter Horses provides an appropriate framework to address these questions because this discipline generates large volumes of repeated performance records, and success is determined by both speed and regularity, which together influence career earnings. Thus, the objectives of this study were to: (1) derive indicators of resilience from longitudinal barrel racing time records in Brazilian Quarter Horses; (2) estimate genetic parameters for these indicators; and (3) quantify the genetic relationships between resilience indicators, barrel racing time, and accumulated career earnings.

## 2 Materials and Methods

### 2.1 Racing Performance Data and Quality Control

Racing performance data for Quarter Horses were obtained from the “Sistema de Gerenciamento de Provas” (SGP) and comprised records from barrel racing events held between 2010 and 2024 across 23 Brazilian states. Longitudinal barrel racing time (BRT) records constituted the primary data source for the present study. Barrel racing is a timed Western rodeo event in which the horse–rider combination completes a prescribed cloverleaf pattern around three barrels. The run begins with the competitor entering the arena at speed and choosing either the right or left barrel to initiate the pattern, followed by the opposite barrel and then the third barrel, before returning through the center line to finish the course. Additional details on data characteristics and the barrel racing circuit are described in Santana et al. (2025).

Barrel racing time was recorded in seconds and defined as the elapsed time required for a horse–rider combination to complete the official three‐barrel cloverleaf course. According to competition regulations, a 5‐s penalty is imposed for each barrel knocked down during a run. As explicit information on penalties was not available in the dataset, a conservative data‐editing procedure was adopted to minimize the inclusion of penalized performances. This procedure was informed by preliminary exploratory analyses and by filtering rules previously described by Santana et al. (2025). Specifically, only BRT observations not exceeding 4.99 s above the Brazilian national record of 16.109 s were retained, thereby removing performances likely influenced by penalties. Additionally, records from races with fewer than ten competing horses were excluded, along with records from horses lacking sire or dam information and from horses with fewer than six BRT observations. Further data quality control involved excluding records from horses younger than 36 months or older than 144 months at the time of competition, restricting the analysis to a biologically and competitively relevant age interval. Finally, rider effects were accounted for by removing records associated with riders who competed in fewer than three races, reducing potential bias from sparsely represented horse–rider pairings. After quality control, 334 508 BRT records from 10 665 horses were retained in the final dataset (Table 1).

**TABLE 1:** Summary of the data structure for barrel racing time (BRT, s) for Brazilian Quarter Horses.

Statistics	BRT
Horses in the pedigree file, *n*	31 639
Records, *n*	334 508
Horses with records, *n*	10 665
Equivalent complete generations (horses with records), *n*	7.89
Mean number of records per horse, *n*	31.36
Horses with up to 10 records, *n*	2764
Horses with 11 to 20 records, *n*	2829
Horses with 21 to 30 records, *n*	1579
Horses with 31 to 40 records, *n*	964
Horses with 41 to 50 records, *n*	655
Horses with more than 50 records, *n*	1874
Male records, *n*	164 163
Female records, *n*	170 345
Stallions with progeny record, *n*	1631
Mares with progeny record, *n*	5621
Contemporary groups, *n*	12 231
Riders, *n*	4693
Mean and standard deviation of the trait	17.939; 0.712
Minimum and maximum of the trait	16.109; 21.098

### 2.2 Resilience Indicators Derived From Race Performance

To derive resilience indicators from longitudinal barrel racing performance, a two‐step modeling strategy was adopted to separate systematic sources of variation from persistent horse‐ and rider‐specific performance levels. Although all effects could, in principle, be fitted simultaneously in a single mixed model, the high‐dimensional contemporary group structure and the large number of horse and rider levels made the two‐step procedure more computationally stable.

First, BRT records were pre‐adjusted for systematic non‐genetic effects using a linear model fitted with the fixest package in R. This model included contemporary group (event identification, competitive class, and competition date) and sex effects, as well as linear and quadratic effects of horse age at competition (in months). Residuals from this model represented BRT observations adjusted for known systematic sources of variation.

In the second step, these pre‐adjusted residuals were analyzed using a linear mixed model fitted with the lme4 package in R. This model included random intercepts for horse and rider to remove persistent phenotypic differences in average performance and rider‐specific effects. Conditional residuals from this model were interpreted as short‐term deviations around each horse's expected performance level after accounting for both systematic and persistent sources of variation. These residual deviations formed the basis for the calculation of resilience indicators.

For each horse, the residual deviations obtained from the pre‐adjustment model were summarized across all available repeated competition records to derive three horse‐level resilience indicators. Specifically, the median absolute deviation (MAD) was calculated as a measure of the magnitude of within‐horse performance fluctuations. The logarithm of the residual variance (LNVAR) was used to quantify overall dispersion of deviations, reflecting the extent of performance variability across events. In addition, the proportion of positive deviations (PPOS) was calculated as the fraction of residuals exceeding zero (worse performance), capturing asymmetry in the direction of deviations relative to the expected performance trajectory. Accordingly, for all three resilience indicators derived in this study, lower values indicate greater resilience. Other commonly used indicators, such as autocorrelation and skewness, were not evaluated because they have been previously reported to be sensitive to recording frequency or to be less reliable for ranking animals in terms of resilience (Zefreh et al. 2024). In the present study, BRT records were competition‐based rather than collected at fixed time intervals. Thus, the interval between consecutive records varied among horses and across career stages according to each animal's competition schedule.

For horses with valid BRT records, accumulated career earnings (ACE), expressed in Brazilian Real (BRL), were retrieved. Preliminary analyses indicated that this trait was not normally distributed; therefore, a logarithmic transformation was applied to the phenotypic values, consistent with previous studies on earnings‐related traits (Thiruvenkadan et al. 2009; Velie et al. 2015). Descriptive statistics for resilience indicators and ACE are presented in Table 2.

**TABLE 2:** Descriptive statistics for resilience indicators derived from barrel racing time records and accumulated career earnings (ACE) in Brazilian Quarter Horses.

Trait	*N*	Mean	Median	SD	Minimum	Maximum	CV (%)
MAD	10 665	0.262	0.244	0.097	0.019	0.976	37.070
LNVAR	10 665	−1.880	−1.903	0.604	−5.810	0.456	32.128
PPOS	10 665	0.473	0.467	0.093	0.100	0.857	19.589
ACE (BRL)	10 665	14 720	5520	29 515	1.000	784 169	200.517
ACE (log)	10 665	8.298	8.616	2.232	0.000	13.572	26.897

Abbreviations: BRL = Brazilian Real, CV = coefficient of variation, LNVAR = logarithm of variance, MAD = mean absolute deviation, PPOS = proportion of positive deviations, SD = standard deviation.

### 2.3 Genetic Analysis

(Co)variance components were estimated using a four‐trait animal model including the three resilience indicators and ACE. Based on preliminary analyses, systematic effects of birth year and sex were included in the model, along with the number of race starts fitted as both linear and quadratic covariates. In addition, for ACE, career length (in years, ranging from 1 to 9) and horse age at first competition (linear covariate) were included. For all traits, random additive genetic and residual effects were included in the model.

To further investigate the genetic relationships between resilience indicators and BRT, an additional four‐trait model was initially evaluated, combining repeated BRT records with single observations of the resilience indicators. However, convergence issues were encountered for this analysis. To address this limitation, (co)variance components for BRT were successfully estimated using a single‐trait repeatability animal model. In this model, contemporary group (as described above), sex, and horse age at competition (in months) were included as systematic effects, with age fitted as both linear and quadratic covariates. Random effects for rider, additive genetic, permanent environment, and residual were also included. Estimates obtained from this model were subsequently used to calculate correlations between estimated breeding values for BRT and the resilience indicators, as detailed in the following section.

All analyses were conducted under a Bayesian framework. Markov chain Monte Carlo sampling consisted of 450 000 iterations, with a burn‐in period of 100 000 iterations and a thinning interval of 25, resulting in 14 000 posterior samples used for inference. Convergence and mixing of the chains were visually assessed through inspection of trace plots. Default priors implemented in the BLUPF90 family of programs were adopted for all analyses, assuming flat priors for systematic effects and inverse‐Wishart priors for (co)variance components. Estimation of (co)variance components and genetic parameters was performed using the GIBBSF90+ program (Misztal et al. 2014).

### 2.4 Genetic Relationships Between Resilience Indicators and BRT and Partial Genetic Correlations

Pearson correlations between estimated breeding values (EBV) for BRT and EBV for the resilience indicators were computed as proxies for genetic correlations. In this analysis, only EBV with accuracy greater than 0.40 were considered for all traits.

Because BRT may partly mediate the genetic association between resilience indicators and ACE, partial genetic correlations were subsequently estimated. Partial genetic correlations quantify the genetic association between resilience indicators and ACE after accounting for differences in genetic merit for BRT, that is, among horses with the same BRT level. Partial genetic correlations were calculated as follows:

$$r_{\mathrm{xy},z}=\frac{r_{\mathrm{xy}}-r_{\mathrm{xz}}r_{\mathrm{yz}}}{\sqrt{\left(1-r_{\mathrm{xz}}^{2})(1-r_{\mathrm{yz}}^{2}\right)}}$$

where $r_{\mathrm{xy}}$ denotes the genetic correlation from multiple trait model between EBV for the resilience indicator (x) and EBV for ACE (y), $r_{\mathrm{xz}}$ represents the Pearson correlation between EBV for the resilience indicator and EBV for BRT (z), and $r_{\mathrm{yz}}$ corresponds to the Pearson correlation between EBV for ACE and EBV for BRT. This approach allows the genetic association between resilience indicators and economic performance to be evaluated independently of genetic differences in competitive speed.

### 2.5 Genetic Ranking and Phenotypic Differences Associated With Resilience

As an initial descriptive assessment, the dispersion of observed BRT records across the competitive lifespan was visualized for the 20 most resilient and the 20 least resilient horses selected based on EBV (accuracy ≥ 0.40) for each resilience indicator. This graphical comparison was used to illustrate differences in phenotypic variability between genetically divergent resilience groups.

To further characterize the genetic separation underlying these comparisons, animals were ranked separately according to their EBV for each resilience indicator. Based on these rankings, the 200 most resilient and the 200 least resilient horses were selected, considering only animals with EBV accuracy of at least 0.40. For each indicator and group, the mean EBV and the corresponding mean accuracy were calculated. These summaries were used to confirm the genetic contrast between the top and bottom resilience groups before evaluating their phenotypic profiles.

Phenotypic differences associated with genetic resilience ranking were then assessed by retrieving BRT and ACE records from the original dataset for the same top and bottom resilience groups. Mean and median values of BRT and ACE were calculated separately for the most and least resilient groups within each indicator. Differences between these summary statistics were subsequently computed to describe phenotypic contrasts associated with divergent genetic resilience profiles.

## 3 Results

### 3.1 Data

Descriptive statistics for the resilience indicators and ACE are shown in Table 2 for 10 665 horses. The resilience indicators derived from BRT showed moderate dispersion. MAD ranged from 0.019 to 0.976 and exhibited the highest relative variability among the indicators (CV = 37.1%), whereas LNVAR ranged from −5.810 to 0.456 (CV = 32.1%). PPOS displayed a narrower distribution, varying from 0.100 to 0.857, with the lowest relative variability (CV = 19.6%). ACE on the original scale showed substantial dispersion and strong skewness, with a wide range (1 to BRL 784 169) and a high coefficient of variation (200.5%). Log‐transformed ACE exhibited reduced dispersion, with a coefficient of variation of 26.9%.

### 3.2 Genetic Parameters

Posterior mean heritability estimates for the resilience indicators were generally low, as shown in Table 3. Heritability for MAD was estimated at 0.072, with an HPD95% interval ranging from 0.045 to 0.106. LNVAR exhibited a slightly higher heritability estimate of 0.092. PPOS showed the lowest heritability among the resilience indicators, with a posterior mean of 0.063 and an HPD95% interval spanning from 0.031 to 0.100. ACE presented a heritability estimate of 0.081, with an HPD95% interval from 0.049 to 0.124. For BRT, the rider effect accounted for a considerable proportion of the phenotypic variance, with a posterior mean of 0.245 (0.235 to 0.255). Heritability of BRT was estimated at 0.207, with an HPD95% interval ranging from 0.186 to 0.230. Repeatability of BRT reached 0.397, indicating that a substantial proportion of the phenotypic variance was associated with effects persistent across repeated records. Posterior standard deviations for BRT‐related parameters were small, and HPD95% intervals were narrow.

**TABLE 3:** Posterior means, standard deviations (SD), and 95% highest posterior density intervals (HPD95%) for heritability, rider effect as a proportion of the phenotypic variance, and repeatability of resilience indicators, barrel racing time (BRT), and accumulated career earnings (ACE) in Brazilian Quarter Horses.

Trait	Parameter	Mean	SD	HPD95%
MAD	Heritability	0.072	0.015	0.045 to 0.106
LNVAR	Heritability	0.092	0.023	0.053 to 0.143
PPOS	Heritability	0.063	0.018	0.031 to 0.100
ACE	Heritability	0.081	0.019	0.049 to 0.124
BRT	Rider	0.245	0.005	0.235 to 0.255
BRT	Heritability	0.207	0.011	0.186 to 0.230
BRT	Repeatability	0.397	0.005	0.387 to 0.406

Abbreviations: LNVAR = logarithm of variance, MAD = mean absolute deviation, PPOS = proportion of positive deviations.

### 3.3 Genetic Correlations Among Resilience Indicators and Accumulated Career Earnings

Posterior mean genetic correlations among the resilience indicators and ACE obtained from the multiple‐trait animal model are summarized in Table 4. A very strong genetic relationship was observed between MAD and LNVAR, with a posterior mean correlation of 0.987, indicating near‐complete overlap in the genetic information captured by these two indicators. Genetic associations involving PPOS were comparatively weaker but remained of moderate magnitude, with posterior means of 0.546 for MAD–PPOS and 0.453 for LNVAR–PPOS, accompanied by broader HPD95% intervals.

**TABLE 4:** Posterior means, standard deviations (SD), and 95% highest posterior density intervals (HPD95%) for genetic correlations obtained from a multiple‐trait analysis of resilience indicators and accumulated career earnings (ACE) in Brazilian Quarter Horses. Partial genetic correlations ($r_{\mathrm{xy},z}$) were calculated between resilience indicators and ACE.

Traits	Mean	SD	HPD95%	$r_{\mathrm{xy},z}$
MAD—LNVAR	0.987	0.006	0.973 to 0.995	—
MAD—PPOS	0.546	0.162	0.207 to 0.837	—
MAD—ACE	−0.671	0.150	−0.899 to −0.344	−0.604
LNVAR—PPOS	0.453	0.199	0.176 to 0.702	—
LNVAR—ACE	−0.564	0.101	−0.755 to −0.282	−0.491
PPOS—ACE	−0.539	0.123	−0.740 to −0.284	−0.376

Abbreviations: LNVAR = logarithm of variance, MAD = mean absolute deviation, PPOS = proportion of positive deviations, x = resilience indicator, y = ACE, z = barrel racing time.

Genetic correlations between the resilience indicators and ACE were consistently negative (favorable) and of moderate magnitude. MAD showed the strongest association with ACE, with a posterior mean of −0.671, whereas LNVAR and PPOS exhibited similar negative correlations, with posterior means of −0.564 and −0.539, respectively. After accounting for genetic differences in BRT, partial genetic correlations between resilience indicators and ACE were slightly reduced in magnitude while remaining favorable. Partial correlations ranged from −0.604 for MAD to −0.376 for PPOS, with LNVAR presenting an intermediate value (−0.491). These estimates indicate that a small part of the genetic association between resilience indicators and economic performance was shared with genetic differences in competitive speed.

### 3.4 EBV‐Based Associations of Resilience Indicators and ACE With BRT

Pearson correlations between estimated breeding values for resilience indicators, ACE, and BRT are presented in Table 5 Favorable positive correlations of moderate magnitude were observed between the resilience indicators and BRT. MAD showed a correlation of 0.386 with BRT, whereas LNVAR exhibited a slightly lower association. The strongest favorable association with BRT was observed for PPOS, with an estimated correlation of 0.594.

**TABLE 5:** Pearson correlations between estimated breeding values, 95% confidence intervals (95% CI) and *p*‐value for resilience indicators, accumulated career earnings (ACE) and barrel racing time (BRT) in Brazilian Quarter Horses.

Traits	Estimate	CI95%	*p* ^a^
MAD—BRT	0.386	0.371 to 0.400	< 0.0001
LNVAR—BRT	0.336	0.321 to 0.351	< 0.0001
PPOS—BRT	0.594	0.583 to 0.604	< 0.0001
ACE—BRT	−0.452	−0.465 to −0.438	< 0.0001

Abbreviations: LNVAR = logarithm of variance, MAD = mean absolute deviation, PPOS = proportion of positive deviations.

^a^ The null hypothesis assumes that the true correlation is equal to zero.

The relationship between ACE and BRT was negative and favorable, with an estimated correlation of −0.452, indicating that lower BRT values were associated with higher genetic merit for economic performance. For all trait pairs, confidence intervals were narrow and clearly separated from zero, and all correlations were statistically significant based on Pearson tests.

### 3.5 Phenotypic Patterns Associated With Resilience Ranking

The EBV distributions confirmed clear genetic divergence between the top and bottom resilience groups for all indicators. For MAD, mean EBV values were −0.028 and 0.028 for the top and bottom groups, respectively. For LNVAR, the corresponding values were −0.183 and 0.191, whereas for PPOS they were −0.027 and 0.018. No overlap was observed between the EBV ranges of the top and bottom groups for any indicator. Mean EBV accuracies were moderate and similar between top and bottom groups for MAD (0.539 vs. 0.524) and LNVAR (0.521 vs. 0.520), whereas for PPOS they were 0.547 and 0.477, respectively.

**FIGURE 1:**
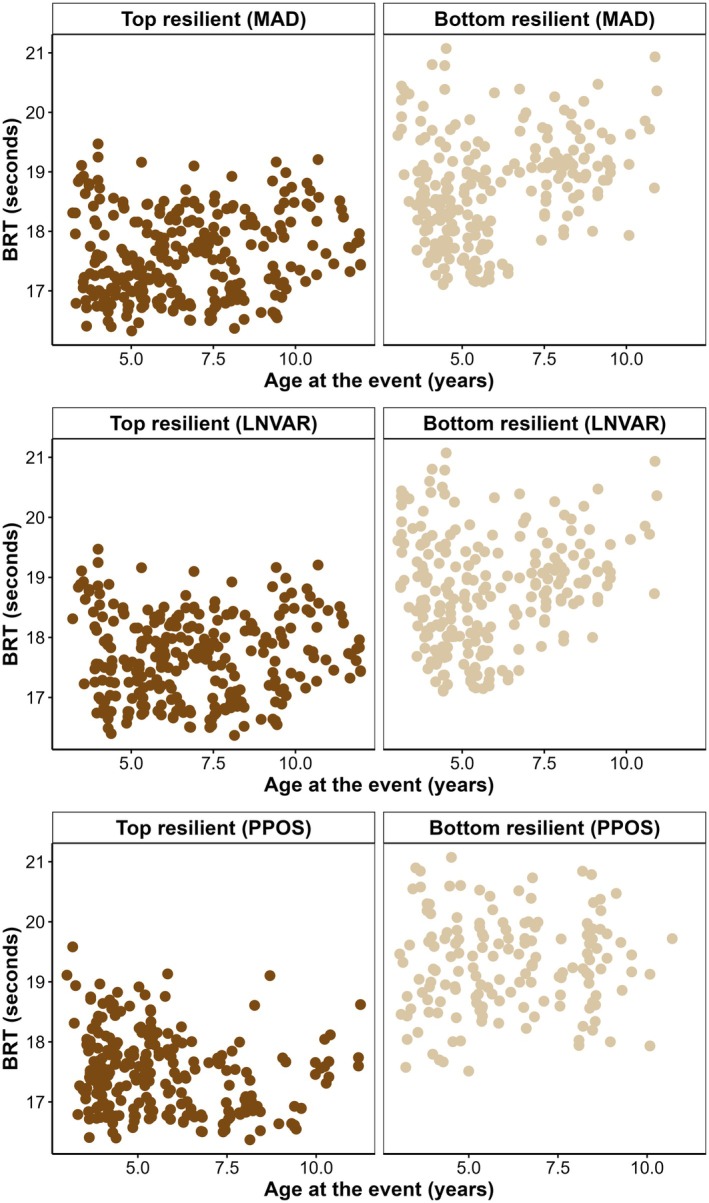
Dispersion of longitudinal barrel racing time (BRT) records for 20 horses classified as highly resilient and less resilient based on estimated breeding values for each resilience indicator: Median absolute deviation (MAD), logarithm of the variance of deviations (LNVAR), and proportion of positive deviations (PPOS).

Clear differences in the dispersion of observed phenotypic records were observed between horses classified as highly resilient and those classified as less resilient based on EBV for each resilience indicator (Figure 1). For all indicators, the two groups displayed distinct patterns of phenotypic variability. Horses classified among the top 20 for resilience showed a narrower distribution of BRT records across competitions, whereas horses classified among the bottom 20 showed a broader distribution of BRT records across events.

**FIGURE 2:**
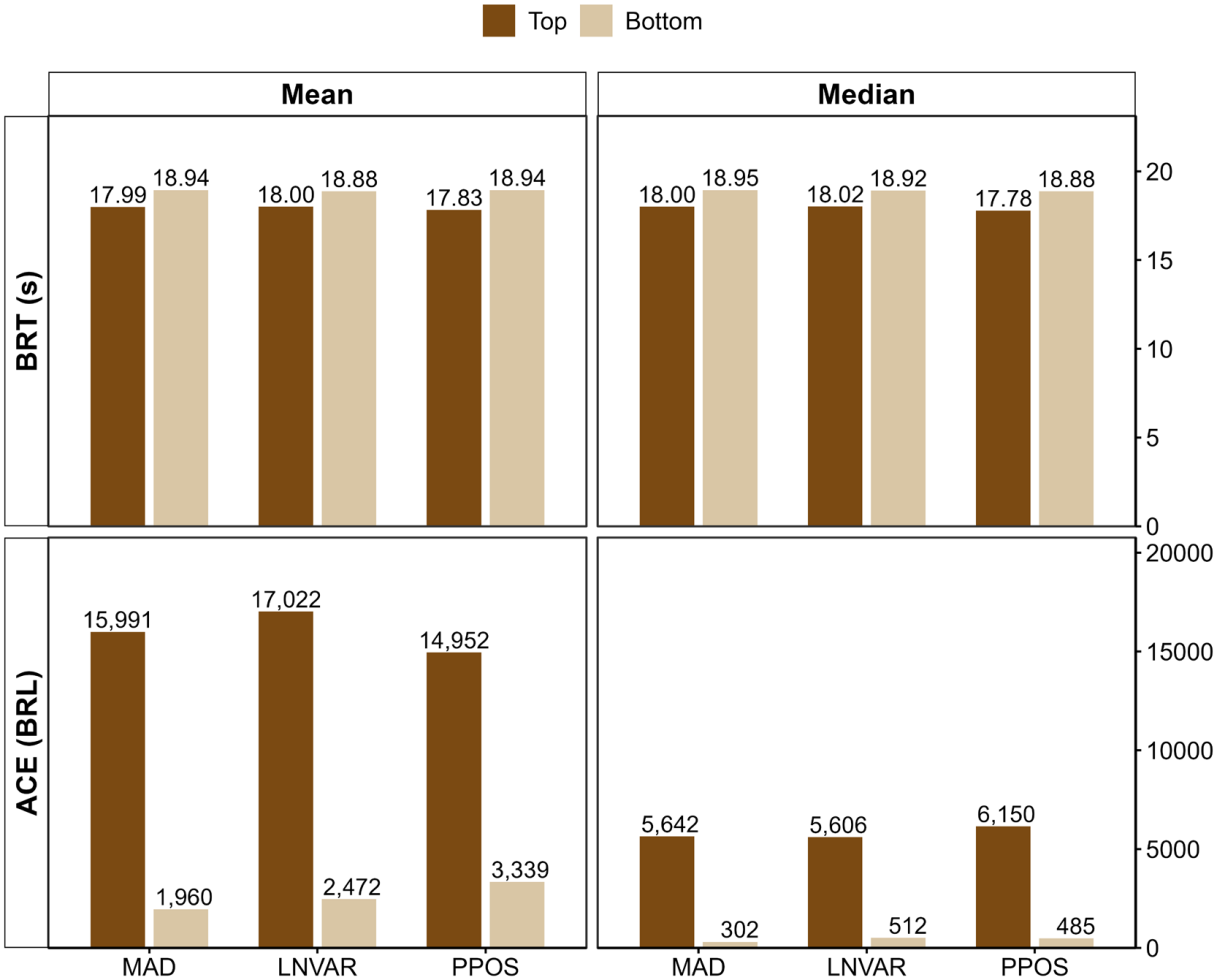
Mean and median barrel racing time (BRT, seconds) and accumulated career earnings (ACE, Brazilian Real) for horses classified among the top 200 and bottom 200 individuals based on estimated breeding values for each resilience indicator: Mean absolute deviation (MAD), logarithm of the variance of deviations (LNVAR), and proportion of positive deviations (PPOS).

Differences in both competitive performance and economic outcomes were observed between horses classified as highly resilient (top 200) and those classified as less resilient (bottom 200) based on EBV for each resilience indicator (Figure 2). For all indicators, horses ranked as more resilient consistently exhibited lower mean and median BRT values compared with those ranked as less resilient. Differences in BRT were evident across indicators, with top‐ranked animals showing mean BRT values close to 18.0 s or lower, whereas bottom‐ranked animals exhibited mean BRT values approaching or exceeding 18.9 s. Consistent patterns were also observed for ACE. For MAD and LNVAR, top‐ranked horses showed mean ACE values exceeding BRL 15 000, whereas bottom‐ranked horses exhibited mean values below BRL 2500. Similar contrasts were observed for PPOS, with top‐ranked horses presenting the highest ACE levels and bottom‐ranked horses showing markedly lower economic returns.

## 4 Discussion

The results of this study provide the first quantitative genetic evidence that resilience, expressed as the stability of competitive performance over time, can be evaluated in sport horses. By deriving resilience indicators from longitudinal barrel racing records and integrating them into a genetic framework, this work extends resilience research beyond traditional livestock production systems to an athletic context characterized by repeated performances under heterogeneous and often unpredictable conditions.

Although resilience indicators in this study were derived from short‐term deviations around an expected performance level, alternative definitions of the underlying performance baseline have been proposed for longitudinal data. In particular, quantile regression has been used to describe a non‐perturbed trajectory as in Poppe et al. (2020). However, when this approach was applied to the present dataset, the resulting resilience indicators showed substantially lower heritability estimates (results not shown) than those obtained with the adopted two‐step modeling approach. This occurred likely because the competition records were irregularly spaced and the number of repeated observations per horse was limited compared with other high‐frequency longitudinal datasets. Under these conditions, an individual quantile‐regression curve may fit not only the expected performance trajectory, but also part of the short‐term fluctuations that resilience indicators are intended to capture. As a result, the residual deviations may contain less between‐horse variation and consequently less genetic signal. In contrast, the adopted two‐step approach, which involved pre‐adjustment for systematic effects followed by the removal of persistent horse‐ and rider‐specific performance levels, provided a more parsimonious definition of the expected performance level while preserving short‐term deviations around that level. This rationale was consistent with the deviation‐based framework described by Berghof, Bovenhuis, et al. (2019), who cautioned that flexible trajectory modeling may absorb biologically meaningful fluctuations when the number of repeated observations per individual is limited.

The genetic parameter estimates indicate that the proposed resilience indicators capture a detectable additive genetic component. Heritability estimates for MAD, LNVAR, and PPOS were consistently low but different from zero, consistent with the fact that these indicators quantify within‐individual variability that is strongly influenced by transient environmental effects. The similar magnitude of heritability across indicators suggests comparable genetic control across different dimensions of performance stability, despite differences in statistical definition. Low heritability for resilience‐related traits has also been reported in livestock species, including dairy cattle (1% to 15%; Chen et al. 2023), pigs (body weight: 2.9% to 20.2%; feed intake: 9.4% to 23.3%; feeding behavior: 16.2% to 28.3%; Gorssen et al. 2023), and layer chickens (9% to 11%; Berghof, Bovenhuis, et al. 2019). Despite these relatively low estimates, there is broad agreement across studies that resilience‐related traits retain sufficient additive genetic variance to respond to selection. In contrast, BRT exhibited a substantially higher heritability, together with moderate repeatability. The heritability estimate obtained in the present study is consistent with values previously reported for the same population by Santana et al. (2025), using both repeatability and random regression models (0.15 to 0.24).

Within this broader context of performance evaluation, ACE represents a trait of relevance in equine breeding, as monetary prizes received by horses and their relatives have historically influenced breeders' selection decisions. Earnings‐related traits are known to be heritable, enabling genetic improvement across different horse populations (Chin et al. 2025; Faria et al. 2023; Velie et al. 2015). In the present study, the results confirm that, despite the limited magnitude of genetic variation, selection for ACE in barrel racing horses can lead to cumulative genetic gains over the long term.

Beyond the magnitude of heritability estimates for candidate resilience indicators, evaluating their genetic relationships with other indicators and with traits of practical relevance is essential. The very strong genetic correlation between MAD and LNVAR indicates an almost complete overlap in their underlying genetic control, suggesting that both indicators largely capture the same genetic dimension of performance variability in Quarter Horses. Similar patterns have been reported in other species, including pigs, where LNVAR showed strong genetic correlations with alternative resilience indicators such as the logarithm of mean squared error (0.93; Gorssen et al. 2023), and in sheep, where genetic correlations exceeding 0.5 were observed between log‐transformed variance and absolute deviation measures derived from fiber diameter and body weight records (Smith et al. 2025).

In contrast, genetic correlations involving PPOS were of moderate magnitude when paired with MAD and LNVAR, indicating that, although PPOS is genetically related to the other indicators, it captures partially distinct aspects of performance resilience. This is consistent with its statistical definition, which emphasizes directional asymmetry in deviations rather than overall dispersion. Nevertheless, the genetic correlations among the resilience indicators suggest that selection for any one of them is expected to result in favorable correlated responses in the others. As suggested by Chen et al. (2024) and Keßler et al. (2025), indicators identified as more promising, particularly those with higher heritability estimates and more favorable genetic correlations with traits of interest, could be combined within a selection index to optimize genetic improvement.

Genetic correlations between the resilience indicators and ACE were consistently favorable and of moderate magnitude. The strongest association was observed between MAD and ACE, followed by LNVAR and PPOS, indicating that horses genetically predisposed to lower performance variability also tended to achieve higher accumulated career earnings. This pattern is consistent with previous findings in Quarter Horses reported by Faria et al. (2023), who documented meaningful genetic relationships between earnings and performance traits, including best time and time class, with negative genetic correlations between earnings and time‐based traits. Together, these results indicate that economic success in barrel racing competitions is genetically associated with superior and more consistent athletic performance rather than with isolated peak outcomes. The similarity in magnitude across these correlations suggests a coherent genetic relationship between resilience‐related traits and long‐term economic performance, while the absence of near‐unity correlations indicates that resilience indicators are not redundant proxies for ACE.

Pearson correlations derived from EBV further indicated favorable positive associations of moderate magnitude between the resilience indicators and BRT, with PPOS showing the strongest association, followed by MAD and LNVAR. In addition, the negative and favorable correlation between ACE and BRT indicates that genetic merit for faster racing times was associated with higher accumulated career earnings. These EBV‐based relationships are consistent with the genetic correlation patterns described above, reinforcing the linkage between competitive efficiency and economic outcomes in barrel racing horses. Although no previous studies have explicitly evaluated the genetic relationships between resilience indicators and performance traits in sport horses, evidence from livestock species indicates that resilience‐related indicators are genetically associated with improved health, longevity, survival, and fertility (Chen et al. 2023; Poppe et al. 2020; Rodrigues et al. 2025). As discussed by Colditz and Hine (2016) and Friggens et al. (2024), such associations reflect fundamental biological properties underlying an animal's capacity to cope with environmental perturbations over time.

The phenotypic contrasts observed between horses classified at opposite extremes of genetic resilience provide a coherent illustration of how resilience is expressed at the performance level. Horses identified as more resilient consistently exhibited reduced dispersion of BRT records across their competitive lifespan, reflecting greater regularity of performance under varying competition conditions. In contrast, less resilient horses showed a wider phenotypic spread, indicating heightened sensitivity to environmental perturbations. These differences in variability were accompanied by phenotypic patterns aligned with the genetic results, including faster average racing times and substantially higher accumulated career earnings among more resilient horses.

The indicators proposed in the present study should be interpreted as data‐driven summaries of performance resilience under competitive conditions. They reflect cumulative deviations across the competitive lifespan, integrating responses to multiple, largely unobserved environmental, management, and physiological challenges. While this approach does not disentangle resistance and recovery processes at a mechanistic level, it enables large‐scale application within the practical constraints of equine sport systems. A limitation of the two‐step procedure is that uncertainty from the pre‐adjustment stage was not fully propagated to the genetic analysis of the derived resilience indicators, a concern also noted for residual‐based approaches to modeling residual variance (Rönnegard et al. 2013). Nevertheless, this strategy provided a computationally feasible framework for deriving resilience phenotypes from sparse and irregular competition records. Consistent with the classification outlined by Taghipoor et al. (2023) and supported by empirical evidence from Berghof et al. (2024), Berghof, Bovenhuis, et al. (2019) and Zefreh et al. (2024), the present methodology falls within the category of data‐driven, post‐treatment resilience quantification. Future advances in this field may arise from new metrics and modeling strategies tailored to sparse and irregular performance records, potentially combined with auxiliary information on perturbation events such as injuries, thermal stress (Santana et al. 2026), or penalized performances, which were not explicitly modeled in the indicators evaluated here.

From a breeding‐program perspective, the findings of the present study suggest that resilience indicators may provide complementary information to traditional selection criteria based on racing time and earnings. Because MAD and LNVAR showed strong genetic similarity, the inclusion of both indicators in routine evaluations may be unnecessary; one dispersion‐based indicator would likely be sufficient to represent genetic differences in performance stability. In contrast, PPOS may add complementary information because it reflects the frequency of unfavorable deviations rather than the overall magnitude of variability. Therefore, a practical implementation could involve evaluating resilience together with BRT and ACE in a selection index, allowing breeders to identify horses that combine faster performance, greater consistency across competitions, and superior long‐term economic outcomes.

## 5 Conclusions

This study demonstrates that performance resilience in sport horses can be quantified using genetic indicators derived from longitudinal competition records. Resilience indicators developed from barrel racing time trajectories in Brazilian Quarter Horses captured a detectable, although limited, additive genetic component. These indicators also revealed shared and complementary dimensions of performance stability, with substantial overlap between MAD and LNVAR and distinct information provided by PPOS. Favorable genetic relationships with competitive performance and accumulated career earnings indicate that genetically more resilient horses tend to perform faster and achieve greater economic success. The indicators evaluated here provide genetically meaningful information that complements traditional performance traits and offer a scalable approach for incorporating performance resilience into genetic evaluations of sport horses. Our results open new perspectives for equine breeding programs by extending selection objectives beyond peak performance toward improved resilience and long‐term stability of athletic outcomes.

## Author Contributions

**Mário Luiz Santana:** conceptualization, methodology, software, investigation, data curation, formal analysis, writing – original draft, writing – review and editing. **Annaiza Braga Bignardi:** methodology, software, investigation, validation, supervision, writing – review and editing.

## Funding

The authors have nothing to report.

## Conflicts of Interest

The authors declare no conflicts of interest.

## Data Availability

The data that support the main findings of this study are available in Figshare at https://doi.org/10.6084/m9.figshare.32400267.
